# Factors Associated With Involuntary Psychiatric Hospitalization of Youths in China Based on a Nationally Representative Sample

**DOI:** 10.3389/fpsyt.2020.607464

**Published:** 2020-12-03

**Authors:** Feng Geng, Feng Jiang, Rachel Conrad, Tingfang Liu, Yuanli Liu, Huanzhong Liu, Yi-lang Tang

**Affiliations:** ^1^Affiliated Psychological Hospital of Anhui Medical University, Hefei, China; ^2^Hefei Fourth People's Hospital, Hefei, China; ^3^Anhui Mental Health Center, Hefei, China; ^4^Department of Psychiatry, Chaohu Hospital of Anhui Medical University, Hefei, China; ^5^Institute of Health Yangtze River Delta, Shanghai Jiao Tong University, Shanghai, China; ^6^Brigham and Women's Hospital, Boston, MA, United States; ^7^Harvard Medical School Center for Bioethics, Boston, MA, United States; ^8^Institute for Hospital Management of Tsinghua University, Beijing, China; ^9^School of Public Health, Chinese Academy of Medical Sciences and Peking Union Medical College, Beijing, China; ^10^Department of Psychiatry and Behavioral Sciences, Emory University, Atlanta, GA, United States; ^11^Mental Health Service Line, Atlanta VA Medical Center, Decatur, GA, United States

**Keywords:** involuntary hospitalization, psychiatric, youths, China, national survey data

## Abstract

**Objective:** This nationally representative sample investigates demographic, diagnostic and clinical features associated with both voluntary and involuntary psychiatric hospitalization among children and adolescents psychiatrically hospitalized in China.

**Method:** As part of an official national survey, 41 provincial tertiary psychiatric hospitals in China were selected. Data from 196 children and adolescents who were discharged from these psychiatric hospitals from March 19 to 31, 2019 were retrieved and analyzed.

**Results:** 1. Psychotic symptoms, depressive symptoms and self-injury/suicide were the most common reasons of admission. Girls were significantly likely to be admitted due to depressive symptoms, whereas boys were more likely to be admitted due to aggressive behaviors. 2. The overall rate of involuntary admission was 32.1% (*N* = 63). Compared to patients who were admitted voluntarily, those who were admitted involuntarily had lower GAF scores on admission, were older, were more likely to present with psychotic symptoms, manic symptoms or aggressive behavior as primary reason for admission, were less likely to present with depressive symptoms, had a significantly longer length of stay, were more likely to be diagnosed with schizophrenia and were less likely to be diagnosed as depressive disorder. 3. A logistic regression showed that depressive symptom as primary reason for admission was significantly associated with voluntary admission (OR = 0.159, *p* < 0.001), along with two other factors: age (*p* < 0.01) and a lower GAF score at admission (*p* < 0.001) were significantly associated with involuntary admission.

**Conclusion:** The rate of involuntary psychiatric hospitalization among children and adolescents is higher in China than in other regions. Developing more specific and more operational criteria to guide involuntary psychiatric admission for child and adolescent patients is of urgency and great importance to ensure appropriate treatment of these patients and protect their rights.

## Introduction

Although outpatient treatment in the community is appropriately recommended for most children and adolescents with psychiatric illness (1, 2), inpatient psychiatric care remains a necessary and important treatment option when a more structured and restrictive environment is required due to safety concerns or severely impaired functioning (3). Common cited indications for psychiatric hospitalization among children and adolescents include psychotic symptoms, self-injury, suicidal ideation, suicide attempts and aggressive behavior (4, 5). One recent study of adolescents psychiatrically hospitalized in the United States found that the percentage of psychiatric hospitalizations for suicidal ideation or self-harm doubled during a period of 5 years (6).

As some patients who are suffering from severe psychiatric illness to have poor insight into the urgent need for treatment of their psychiatric illness during a decompensation, most countries have laws which permit involuntary psychiatric hospitalization in certain circumstances (7). A rights-based ethical approach to involuntary psychiatric hospitalization requires that any acts of coercion that violate respect for a patient's autonomy are well justified (8). Various international efforts, including the Principles for the Protection of Persons with Mental Illness, the European Convention for the Protection of Human Rights and Fundamental Freedoms, The Declaration of Hawaii, and the Ten Basic Principles for Mental Health Law published by the World Health Organization aim to provide guidance on the rights of people with psychiatric illness. However, despite efforts to standardize the criteria justifying involuntary psychiatric hospitalizations, there are marked regional disparities in both laws and clinical practices (9). Most laws governing involuntary psychiatric hospitalizations require that the patient demonstrates symptoms of psychiatric illness and involuntary treatment is needed due to safety risk.

In addition to the duty to weigh ethical considerations regarding violating a patient's human rights, involuntary psychiatric hospitalization can experienced by patients as coercive and may have negative long-term effects, including traumatization and decrease future help-seeking. One study showed that 36% of patients who had received coercive treatment including involuntary psychiatric hospitalization would not seek professional help for psychiatric illness because of fear of coercive measures (10).

Previous studies on involuntary psychiatric hospitalizations in psychiatry have primarily involved adult samples (11–13). Due to the unique developmental and clinical features and needs during childhood and adolescence, the findings in the adult population may not be generalizable to the child and adolescent populations. Literature on involuntary psychiatric hospitalization of children and adolescent patients is scarce (14–17). Studies from Finland and Germany found that the involuntary psychiatric hospitalization rate in child and adolescent psychiatric inpatients was between 11 and 29.5%, and involuntary admission was significantly associated with substance use and psychotic disorders (14, 17).

In China, the first national legislation pertaining to involuntary psychiatric treatment was the Mental Health Law of China (MHL), which was passed by the Standing Committee of the National People's Congress of China in 2012 and implemented in 2013. Prior to this law, there was little guidance regarding the appropriate use of involuntary psychiatric hospitalization in China (18). China's MHL addresses many aspects of mental health treatment and aims to provide a legal foundation to protect patients' rights and to guide involuntary psychiatric hospitalization for patients who are at high risk of harming themselves or others due to psychiatric illness (19).

Since MHL took effect on May 1, 2013, a few studies have examined the effects of this law on the adult population (13, 20), but no studies have focused on the effects of this law on child and adolescent populations. Both the prevalence of and the circumstances justifying involuntary psychiatric hospitalization among children and adolescents are important and may help identify gaps in the legislation protecting this vulnerable patient population. As a part of nation-wide survey, we collected data regarding child and adolescent patients discharged from psychiatric hospitals from 41 top-tier psychiatric hospitals in China. We focused on voluntary admission and its demographic and clinical correlates.

## Methods

This study was a part of a large research project, the National Survey for the Evaluation of Psychiatric Hospital Performance (21). One to three provincial psychiatric hospitals under the jurisdiction of the Ministry of Health of each province were selected. These hospitals were under the jurisdiction of the Ministry of Health (now incorporated into the new National Health Commission) in each province. We also did not include hospitals within the jurisdiction of the Ministry of Public Security (Forensic psychiatric hospitals) and the Ministry of Social Welfare (Safety net hospitals) as their patient populations are different and they often follow different guidelines about staffing and resource allocations. Gansu and Tibet were not included because there were no provincial psychiatric hospitals at the time of survey. In total, we selected 41 top-tier psychiatric hospitals from 29 provinces and autonomous regions in Mainland China. We included all children and adolescents under 18 years old who were discharged from March 19 to 31, 2019. Clinicians who were not directly involved in the patients' care interviewed the patients. We excluded 4 patients (2%) who stayed in hospitals for <1 day or those who were missing one or more items. Finally, data from 196 patients were analyzed in this study.

Patients' demographic information and clinical features were collected by site-based research staff using semi-structured interviews and discharge medical records. The retrieved data included age, sex, discharge diagnosis according to International Classification of Diseases and Related Health Problems, 10th revision (ICD-10), length of stay (LOS), voluntary/involuntary treatment, first admission/readmission, main reasons for admission (multiple choices), and whether they had aggressive behaviors, or self-injury/suicide during hospitalization.

The Ethics Committee of Chaohu Hospital of Anhui Medical University (No. 201903-kyxm-02) and each participating hospital approved this study.

Summary statistics were used to describe the data. Comparison of days of hospitalization in various subgroups was calculated using Mann-Whitney U test as appropriate. A Chi-square test was used to compare other factors in various subgroups. The SPSS version 22.0 software (IBM Corp, Armonk, NY) was used to perform the basic statistical analyses.

All the tests were two-sided and statistical significance was defined as *P* < 0.05.

## Results

### General Features of Children and Adolescents Psychiatrically Hospitalized in China

As shown in Table 1, most patients in this sample were 15 years or older. The majority (111/196, 55.6%) of the sample were girls. Nearly 70% (136/196, 69.4%) were first time admissions. Psychotic symptoms, depressive symptoms and self-injury/suicide were the most common reasons of admission. The average length of stay was 32.53 ± 22.42 days (from 1 to 156 days, with a median of 28 days). The most common diagnoses (ICD-10) were schizophrenia (29.6%), depressive disorder (31.1%) and mania/bipolar disorder (16.3%).

**Table 1:** Clinical and demographic features and gender differences of 196 discharged child and adolescent inpatients.

**Variables**	***N* = 196**	**Boys (*n* = 85)**	**Girls (*n* = 111)**	**x^**2**^, z**	***p***
Age (years)	15.28 ± 1.79	15.54 ± 1.67	15.08 ± 1.85	−1.963	0.050
15–17 years old	135 (68.9%)	64 (47.4%)	71 (52.6%)	4.398^a^	0.098
12–14 years old	56 (28.6%)	18 (32.1%)	38 (67.9%)		
Under 12 years old	5 (2.5%)	3 (3.5%)	2 (1.8%)		
First admission	136 (69.4%)	58 (68.2%)	78 (70.3%)	0.094	0.759
**Primary reason of admission**					
Psychotic symptoms	92 (46.9%)	45 (52.9%)	47 (42.3%)	2.171	0.141
Depressive symptoms	84 (43.3%)	28 (32.9%)	57 (51.4%)	6.643	0.010
Manic symptoms	27 (13.8%)	11 (12.9%)	16 (14.4%)	0.088	0.767
Aggressive behaviors	28 (14.3%)	18 (21.2%)	10 (9.0%)	5.820	0.016
Self-injurious and suicidal behaviors	34 (17.3%)	11 (12.9%)	23 (20.7%)	2.032	0.154
Risk of violence	2 (1%)	–	–	–	–
Medication adjustment	19 (9.7%)	–	–	–	–
Poor self-care ability	10 (5.1%)	–	–	–	–
Others	7 (3.6%)	–	–	–	–
Mean length of stay (days)	32.53 ± 22.42	36.25 ± 25.74	29.68 ± 19.13	−1.784	0.061
0–7 days	11 (5.6%)	3 (3.5%)	8 (7.2%)	8.832^a^	0.023
8–30 days	99 (50.5%)	37 (43.5%)	62 (55.9%)		
31–90 days	82 (41.8%)	41 (48.2%)	41 (36.9%)		
More than 90 days	4 (2%)	4 (4.7%)	0 (0.0%)		
Aggressive behaviors during hospitalization	62 (31.6%)	30 (35.3%)	32 (28.8%)	0.930	0.335
Self-injury/suicide during hospitalization	24 (12.2%)	5 (5.9%)	19 (17.1%)	5.654	0.017
**Diagnosis**					
Psychosis	58 (29.6%)	27 (31.8%)	31 (27.9%)	7.282	0.063
Depression	61 (31.1%)	10 (11.8%)	22 (19.8%)		
Bipolar Disorder	32 (16.3%)	22 (25.9%)	39 (35.1%)		
Others	45 (23%)	26 (30.6%)	19 (17.1%)		
Diagnosis as any mood disorder	93 (47.4%)	32 (37.6%)	61 (55.0%)	5.783	0.016

a *Fisher's Exact Test*.

### Gender Differences

No significant gender differences were found in age, distribution of age groups and rate of first hospitalization (all *P*s > 0.05). Girls were significantly more likely to be admitted due to depressive symptoms (51.4 vs. 32.9%, *p* < 0.05), whereas boys were more likely to be admitted due to aggressive behaviors (21.2 vs. 9.0%, *p* < 0.05). There were no significant gender differences on other primary admission reasons. However, during hospital stay, girls were more likely to have self-injury/suicide attempts than boys (17.1 vs. 5.9%, *p* < 0.05).

### Clinical Features of Children and Adolescents Involuntarily Psychiatrically Hospitalized

Overall, sixty-three patients (63/196, 32.1%) were hospitalized involuntarily. There was no significant gender difference. Patients who were admitted involuntarily were significantly older (15.65 ± 1.59 vs. 15.11 ± 1.86, *p* < 0.05), had a significantly lower baseline GAF scores (39.08 ± 19.69 vs. 51.74 ± 16.91, *p* < 0.001). Most (87.3%) of those patients who were involuntary admitted were admitted due to psychotic symptoms, aggressive behaviors and manic symptoms. Among primary reasons of admission, the percentage of psychotic symptoms (63.5 vs. 39.1%, *p* < 0.01) and aggressive behaviors (23.8 vs. 9.8%, *p* < 0.01) were significantly higher among those who were involuntary hospitalized than those who were voluntarily hospitalized, while percentage of depressive symptoms was significantly lower among those who were involuntary hospitalized than those who were voluntarily hospitalized (14.3 vs. 57.1%, *p* < 0.001). Nineteen cases had aggressive or self-injury/suicide behaviors before admission, and four cases had both aggressive and self-injury/suicide behaviors. One patient, a 13 year-old boy, was admitted involuntarily because he refused to go to school. The patients who were admitted involuntarily presented more aggressive behaviors (52.4 vs. 21.8%, *p* < 0.001) but with no significant difference in self-injury/suicide behaviors (14.3 vs. 11.5%, *p* = 0.549) during hospitalization. The patients who were involuntary psychiatrically hospitalized had a significantly longer length of stay than those who were voluntarily hospitalized (38.40 ± 24.27 vs. 29.74 ± 21.01 days, *p* < 0.05). More involuntary patients (57.1 vs. 34.6%) were in the subgroup of 31–90 days, while more voluntary patients (57.9 vs. 34.9%) were in the subgroup of 8–30 days. Involuntary patients were more likely to be diagnosed with schizophrenia (46.0 vs. 21.8%) and less likely to be diagnosed with a depressive disorder (14.3 vs. 39.1%). No significant differences were found in the clinical response rates between the two groups.

### Factors Associated With Involuntary Hospitalization

A logistic regression was performed to assess the association between independent variables and the likelihood of being admitted involuntarily. The model contained seven independent variables: age, GAF scores on admission, primary reasons of admission (psychotic symptoms, depressive symptoms, aggressive behaviors) and diagnosis. Because of their low occurrence or equal distribution, other variables were excluded. The full model containing all predictors was statistically significant (omnibus x^2^ = 56.262, *p* < 0.001), indicating that the model was able to distinguish whether admitted involuntarily or not. The model explained between 25.0% (Cox and Snell *R*^2^) and 34.9% (Nagelkerke *R*^2^) of the variance, and correctly classified 71.9% of all cases.

As shown in Table 2, GAF scores on admission and depressive symptoms as primary reason of admission as independent variables made a highly significant contribution (*p* < 0.001) to the model and age as independent variable made a significant contribution (*p* < 0.01). In other words, the strongest predictor of voluntary admission were depressive symptoms as primary reason of admission with OR = 0.159.

**Table 2:** Associations of independent variables with involuntary admission in child and adolescent inpatients.

	**B**	**S.E**.	**Wald**	**df**	**Odds ratio**	**95% C.I. for odds ratio**	
						**Lower**	**Upper**
Age	0.272	0.115	5.631	1	1.313^*^	1.048	1.664
GAF on admission	−0.034	0.010	11.714	1	0.966^**^	0.947	0.985
**Primary reason of admission**							
Psychotic symptoms (ref. no)	0.107	0.510	0.044	1	1.113	0.410	3.024
Depressive symptoms (ref. no)	−1.837	0.542	11.471	1	0.159^**^	0.055	0.461
Aggressive behaviors (ref. no)	0.486	0.491	0.980	1	1.625	0.621	4.253
Diagnosis (ref. schizophrenia and related disorders)			0.253	3			
Bipolar Disorder	0.114	0.663	0.030	1	1.121	0.306	4.110
Depressive Disorder	−0.190	0.648	0.086	1	0.827	0.232	2.943
Other	0.012	0.520	0.000	1	1.012	0.365	2.801
Constant	−2.867	1.919	2.231	1	0.057		

* *p < 0.01*,

** *p < 0.001*.

## Discussion

This is the first nationwide study focusing on involuntary psychiatric hospitalization of children and adolescents in China.

### General Clinical Characteristics

The majority of patients psychiatrically hospitalized were above age 12 years. The most common reasons for admission were psychotic symptoms and depressive symptoms, and the most common diagnoses at discharge were psychosis, depression and mania/bipolar disorder.

Aggression during hospitalization was common among both boys and girls. The incidence of aggression during hospitalization (31.6%) was higher than the incidence reported in studies from other countries, such as 23.1% in Australia and 21.7% in New Zealand, respectively (22, 23).

The average length of stay of children and adolescents psychiatrically hospitalized in this sample (32.53 days) was markedly longer than the average length of stay reported in United States (10.7 days, data from 2008), shorter than Finland (82.2 days, data from 2011), and similar to the United Kingdom (28 days, data from 2010) (3, 7, 24). However, the average length of stay for psychiatric hospitalizations has progressively decreased over time in most countries, including those listed above.

Girls were much more likely to be hospitalized due to depressive symptoms and to be diagnosed with depression at discharge. Recent meta-analyses have indicated that gender differences in rates of depression emerge earlier than previously understood, with girls being significantly more likely to be diagnosed with depression by age 12 and the odds ratio increases during adolescence (OR 2.37 at age 12 and OR 3.02 at age 13–15) (25). Elevated rates of depressive symptoms and depressive disorder diagnosis in this study were consistent with these findings.

Boys were more likely to have aggressive behaviors as primary reason for admission. However, there was no significant difference in the rate of aggression between boys and girls during hospitalization. Aggressive behavior has previously been reported as the most frequent reason for psychiatric hospitalization among adolescent boys (26). While some studies have found higher rates of aggression during hospitalization among boys than girls, this gender difference has not been found consistently among adolescents (23, 27, 28).

The incidence of self-injury/suicide during hospitalization was higher among girls, which is consistent with prior studies. One study showed that 80% of adolescents psychiatrically hospitalized for self-harm were girls, and other research has indicated that adolescent girls have more frequent suicidal behavior during psychiatric hospitalizations (29, 30).

### Factors Associated With Involuntary Hospitalization

Mental Health Law of China (MHL) has no specific criteria regarding involuntary hospitalization for children and adolescents. The criteria for involuntary admission in China's MHL largely followed the examples of laws passed in the United States (31). They are based primarily on risk, including risk of suicide or self-injury, risk of harming others, or both and which is usually determined by psychiatrists (13). Psychiatrists in China show divergent attitudes toward the use of involuntary psychiatric hospitalization for both adults and children (32).

The involuntary admission rate in our sample (32.1%) was higher than the rates found in studies in European countries (19–29.5%), which have shown decreasing rates of involuntary hospitalization in recent years (7, 14, 17). Our study found that patients hospitalized involuntarily were older, which is consistent with previous studies of involuntary hospitalization among children and adolescents (14, 17). The patients in our study who were involuntary hospitalized presented with a wide range of psychotic symptoms and aggressive behaviors before admission, lower levels of general functioning and discharge diagnoses of psychosis, consistent with previous findings in Europe (14, 17).

Both depressive symptoms and a discharge diagnosis of depression were more common in patients with voluntary hospitalization. Additionally, depressive symptoms as primary reason of admission were significantly associated with low likelihood to be admitted involuntarily (OR = 0.159, *p* < 0.001). Self-injury/suicidal behaviors were not significantly different between two groups. These findings are different from previous reports (33, 34). One research showed suicide attempts and affective disorders were strongly associated with involuntary psychiatric hospitalization among children and adolescents (35).

Somewhat surprisingly, the rates of aggression during hospitalization (21.8 and 52.4% in voluntary and involuntary groups, respectively) were significantly higher than rates of aggression before admission among patients with both voluntary and involuntary hospitalization (9.8 and 23.8%). This could suggest the restrictive environment might have worsened the aggressive behavior and patients “acted out” as a response. It is also possible that some patients became more aggressive after they were exposed to aggressive behaviors of others in the same unit, a phenomenon called the “contagion” effect of violence (36). Better communication among treatment team, patients and their families as well as de-escalation training for staff may be helpful to reduce aggression during hospitalization.

Compared to patients with voluntary hospitalization, patients with involuntary hospitalization had a significantly longer LOS, which is different from the findings in Europe. One study found no significant difference in the LOS between involuntary and voluntary hospitalization, and another found that patients with voluntary hospitalization had a longer LOS (14, 17). These differences may reflect the differences in the respective patient population, mental health care systems, or legal systems governing duration of involuntary commitment, among other factors.

## Limitations

A few limitations of the study need to be mentioned. First, as a retrospective study, we could not verify the diagnosis of each patient. The retrospective nature of this study and the structure of the data collection only allow limited analysis. Second, our survey did not distinguish suicide attempts from non-suicidal self-injury, and thus understanding the respective impact of suicide attempts and self-injury on psychiatric hospitalization was limited. Finally, our study did not have data on other important factors, such as family backgrounds, parents, childhood experience, etc., which might also affect some of the findings.

## Conclusion

Based on a large and representative national dataset, we examined the current status of involuntary admission of child and adolescent patients in China. We found rate of involuntary admission among children and adolescents psychiatrically hospitalized was relatively high (32.1%) in China, and we found that involuntary admission was significantly associated with psychotic symptoms, aggressive behaviors, longer length of stay and the diagnosis of schizophrenia. Although Mental Health Law of China was revised in 2018, it still lacks specific criteria governing involuntary hospitalization of child and adolescent patients. Developing more specific and more operational criteria to guide involuntary admission for child and adolescent patients is of urgency and great importance to ensure appropriate treatment of these patients and protect their rights.

## Data Availability Statement

The raw data supporting the conclusions of this article will be made available by the authors, without undue reservation.

## Ethics Statement

The studies involving human participants were reviewed and approved by The Ethics Committee of Chaohu Hospital of Anhui Medical University (No. 201903-kyxm-02) and each participating hospital approved this study. Written informed consent to participate in this study was provided by the participants' legal guardian/next of kin.

## Author Contributions

FG and FJ had full access to all the data in the study and took responsibility for the integrity of the data and the accuracy of the data analysis. FJ, HL, and Y-lT: concept and design. FG and Y-lT: acquisition, analysis, or interpretation of data. FG, HL, and Y-lT: drafting of the manuscript. FG, FJ, and Y-lT: statistical analysis. FJ and HL: obtained funding. HL and Y-lT: supervision. All authors critical revision of the manuscript for important intellectual content.

## Conflict of Interest

The authors declare that the research was conducted in the absence of any commercial or financial relationships that could be construed as a potential conflict of interest.
